# Effect of colchicine on physiological and biochemical properties of Rhodococcus qingshengii

**DOI:** 10.18699/VJGB-22-69

**Published:** 2022-10

**Authors:** Yu.A. Markova, L.A. Belovezhets, V.N. Nurminsky, I.S. Kapustina, N.V. Ozolina, V.V. Gurina, A.L. Rakevich, A.V. Sidorov

**Affiliations:** Siberian Institute of Plant Physiology and Biochemistry of Siberian Branch of Russian Academy of Sciences, Irkutsk, Russia; A.E. Favorsky Irkutsk Institute of Chemistry of the Siberian Branch of the Russian Academy of Sciences, Irkutsk, Russia; Siberian Institute of Plant Physiology and Biochemistry of Siberian Branch of Russian Academy of Sciences, Irkutsk, Russia; Siberian Institute of Plant Physiology and Biochemistry of Siberian Branch of Russian Academy of Sciences, Irkutsk, Russia; Siberian Institute of Plant Physiology and Biochemistry of Siberian Branch of Russian Academy of Sciences, Irkutsk, Russia; Siberian Institute of Plant Physiology and Biochemistry of Siberian Branch of Russian Academy of Sciences, Irkutsk, Russia; Irkutsk Branch of the Institute of Laser Physics, The Siberian Branch of the Russian Academy of Sciences, Irkutsk, Russia; Siberian Institute of Plant Physiology and Biochemistry of Siberian Branch of Russian Academy of Sciences, Irkutsk, Russia

**Keywords:** Rhodococcus qingshengii, colchicine, biof ilms, fatty acids, membrane microviscosity, Rhodococcus qingshengii, колхицин, биопленки, жирные кислоты, микровязкость мембран

## Abstract

The genus Rhodococcus includes polymorphic non-spore-forming gram-positive bacteria belonging to the class Actinobacteria. Together with Mycobacterium and Corynebacterium, Rhodococcus belongs to the Mycolata group. Due to their relatively high growth rate and ability to form biof ilms, Rhodococcus are a convenient model for studying the effect of biologically active compounds on pathogenic Mycolata. Colchicine was previously found to reduce biof ilm formation by P. carotovorum VKM B-1247 and R. qingshengii VKM Ac-2784D. To understand the mechanism of action of this alkaloid on the bacterial cell, we have studied the change in the fatty acid composition and microviscosity of the R. qingshengii VKM Ac-2784D membrane. Nystatin, which is known to reduce membrane microviscosity, is used as a positive control. It has been found that colchicine at concentrations of 0.01 and 0.03 g/l and nystatin (0.03 g/l) have no signif icant effect on the survival of R. qingshengii VKM Ac-2784D cultivated in a buffered saline solution with 0.5 % glucose (GBSS). However, colchicine (0.03 g/l) signif icantly inhibits biof ilm formation. Rhodococcus cells cultivated for 24 hours in GBSS with colchicine acquire a rounded shape. Colchicine at 0.01 g/l concentration increases C16:1(n-7), C17:0, C20:1(n-9) and C21:0 fatty acids. The microviscosity of the membrane of individual cells was distributed from the lowest to the highest values of the generalized laurdan f luorescence polarization index (GP), which indicates a variety of adaptive responses to this alkaloid. At a higher concentration of colchicine (0.03 g/l) in the membranes of R. qingshengii VKM Ac-2784D cells, the content of saturated fatty acids increases and the content of branched fatty acids decreases. This contributes to an increase in membrane microviscosity, which is conf irmed by the data on the GP f luorescence of laurdan. All of the above indicates that colchicine induces a rearrangement of the Rhodococcus cell membrane, probably in the direction of increasing its microviscosity. This may be one of the reasons for the negative effect of colchicine on the formation of R. qingshengii VKM Ac-2784D biof ilms.

## Abstract (ru)

Род Rhodococcus объединяет полиморфные неспорообразующие грамположительные бактерии, относящиеся к классу Actinobacteria. Rhodococcus вместе с Mycobacterium и Corynebacterium входят в группу Mycolata. Благодаря относительно высокой скорости роста и способности к образованию биопленок, Rhodococcus являются удобной моделью для изучения действия биологически активных соединений на патогенные Mycolata. Ранее было показано, что колхицин угнетал образование биопленок у P. carotovorum ВКМ В-1247 и R. quingshengii ВКМ Ac-2784D. Целью настоящей работы было изучение действия колхицина на жирнокислотный состав и микровязкость мембран Rhodococcus qingshengii ВКМ Ac-2784D для понимания механизма действия этого алкалоида на бактериальную клетку. В качестве положительного контроля использовали нистатин, снижающий микровязкость мембран. Установлено, что колхицин в концентрациях 0.01 и 0.03 г/л и нистатин (0.03 г/л) не оказали существенного влияния на выживаемость R. qingshengii ВКМ Ac-2784D, культивируемого в забуференном физиологическом растворе глюкозы (ЗФРГ). Однако колхицин (0.03 г/л) значительно угнетал образование биопленки. Клетки Rhodococcus, культивируемые в течение суток в ЗФРГ с колхицином, приоб- ретали округлую форму. Колхицин в концентрации 0.01 г/л вызывал увеличение жирных кислот C16:1(n-7), С17:0, C20:1(n-9) и C21:0. Микровязкость мембраны отдельных клеток распределялась от максимально низких до максимально высоких значений показателя обобщенной поляризации флуоресценции лаурдана (GP), что свидетельствует о разнообразии адаптационных ответов на этот алкалоид. При более высокой концентрации колхицина (0.03 г/л) в мембранах клеток R. qingshengii ВКМ Ac-2784D увеличивалось содержание насыщен- ных жирных кислот и падало – разветвленных. Это способствовало увеличению микровязкости мембраны, что подтверждается данными по GP. Таким образом, колхицин индуцирует перестройку клеточной мембраны Rhodococcus, вероятно, в сторону увеличения ее микровязкости, что может быть одной из причин негативного действия колхицина на образование биопленок R. qingshengii ВКМ Ac-2784D.

## Introduction

The Rhodococcus genus includes polymorphic non-sporeforming gram-positive bacteria that belong to the Actinobacteria class. Rhodococcus are frequently met in nature, in particular, in living organisms. Among the key features of these microorganisms is their ability to decompose different organic compounds, including pollutants (PAC, biphenyls, alkanes, etc.) (Szőköl et al., 2014; Li et al., 2016). For this reason, Rhodococcus continue to attract the growing interest as valuable biotech species.

Along with Mycobacterium and Corynebacterium, Rhodococcus relates to the Mycolata group, which is characterized by the presence of mycolic acids on the cell walls (Sutcliffe, 1998). This makes these bacteria more resistant to the toxic compounds such as disinfectants, antibiotics or PAC. Unlike myco- and corynebacteria, Rhodococcus species are mostly non-pathogenic. Therefore, owing to relatively high growth rate and propensity to biofilm formation, the Rhodococcus represent a convenient model to examine the effect of biologically active compounds on pathogenic Mycolata.

The integrity of a microbial cell drastically depends on the membrane. In order to survive in ever-changing environmental conditions and to maintain optimal membrane fluidity, the bacteria change the fatty acid composition of membrane lipids (Dubois-Brissonnet et al., 2016). The cell membrane is the major target of non-polar organic solvent toxicity (De Carvalho et al., 2005). Plant metabolites also affect the membrane via inhibition of the efflux channels activity (Tegos et al., 2002), the content of porin proteins (Abreu et al., 2012), etc.

Previously, we found that the alkaloid colchicine at a concentration of 0.25 g/l suppressed the formation of a biofilm by Pectobacterium carotovorum VKM B-1247 and Rhodococcus qingshengii VKM Ac-2784D species (Bybin et al., 2018). Moreover, no negative effect on the viability of these bacteria was revealed. Colchicine is widely known as an alkaloid that interrupts the tubulin polymerization in eukaryotic cells (Zhang et al., 2018). It is likely that colchicine exhibits a similar effect on the microorganisms, affecting the cytoskeleton and preventing the adhesion of microbial cells (Dubey et al., 2011). However, its influence on microbial cells was poorly studied. All of the above sparked our interest in this compound.

In the present work, we have examined the effect of colchicine on the fatty acid composition and microviscosity of R. qingshengii VKM Ac-2784D membranes.

## Materials and methods

R. qingshengii VKM Ac-2784D strain isolated from the rhizosphere of couch grass (Elytrigia repens (L.) Nevski) growing in the oil-contaminated territory of the Irkutsk region (Russia) was used in the work (Petrushin et al., 2021). The Rhodococcus strain features a good formation of biofilms, and therefore represents a convenient model for their study.

The bacteria were cultivated on BTN-agar (Biotekhnovatsiya, Russia) for 48 h at 26 °C. Then they were transferred to a 0.5 % glucose buffered saline solution (GBSS) and the density of the suspension was adjusted to OD595 0.26–0.33.

The minimum inhibitory concentration (MIC) of colchicine for R. qingshengii VKM Ac-2784D was determined by the limiting dilution method (Guidelines..., 2000).

To evaluate the effect of nystatin and colchicine on growth kinetics and biofilm formation, 150 μl of bacterial suspension was added to the wells of sterile flat-bottom 96-well plates and the optical density was measured on the first, third, and eighth days of cultivation using an iMark plate reader (Bio-Rad, USA) , λ = 595 nm. The plate was washed from loosely attached cells. The precipitate was stained with 1 % crystal violet solution for 45 min. After washing (3 times) to extract the dye, 200 μl of 96 % ethanol was added to the wells. The level of extraction (absorption) of crystalline violet with ethanol was measured using an iMark plate reader (Bio-Rad) at a wavelength of 595 nm in optical density units (OD595). The degree of biofilm formation corresponded to the intensity of dye staining of the wells content (Shaginyan et al., 2007).

Two controls were employed in the work. The first one involved the bacteria cultivated in GBSS without the addition of colchicine. The second control used the bacteria grown in a medium with 0.03 g/l of nystatin (Biosintez, Russia), since nystatin can reduce microviscosity of the cell membranes. Colchicine (Sigma-Aldrich, USA) was applied at concentrations of 0.01 and 0.03 g/l. When plotting the diagrams, the relative optical density in % to the control was used. Cell sizes were assessed using the AxioVision Rel 4.8 software.

To determine the fatty acid composition of the bacterial membrane and the orderliness (microviscosity, fluidity) of its lipid phase, the bacteria were cultivated in the aforementioned media for a day. The membrane lipids orderliness was evaluated by the generalized polarization (GP) of laurdan lipophilic probe fluorescence in each pixel corresponding to the luminescent image domain. To stain the bacteria, 10 μM of a methanolic solution of laurdan (2-(dimethylamino)-6-dodecanoylnaphthalene) (Sigma- Aldrich) was added to each vial. Live stained bacteria were observed using a microscope (laser scanning confocal fluorescent microscope MicroTime 200; PicoQuant GmbH, Germany).

The distribution of GP values was analyzed by visualization with histograms. For each histogram, a theoretical multimodal distribution as a superposition of several normal distributions was plotted (Nurminsky et al., 2017). Next, the parameter fitting of the experimental distributions of bacterial membrane GP values was estimated. The model distribution was a normal distribution or a mixture of distributions and thus consisted of one or more components. Finally, the optimal parameters of the components that were closest to the experimental distribution were selected.

To determine the composition of fatty acids (FA), the bacteria were cultivated similarly without the addition of laurdan. The lipids were extracted according to the published procedure (Bligh, Dyer, 1959). After removal of the solvent, a 1 % methanol solution of H2SO4 was added to the lipid extract and heated on a water bath at 60 °C for 30 min. After cooling, the solution was extracted (3 times) with hexane (Christie, 1993). Fatty acids methyl esters were analyzed using an Agilent technology 5973N/6890N MSD/ DS chromato-mass spectrometer (USA). Detector (mass spectrometer) was quadrupole, ionization method was electron impact (EI), ionization energy was 70 eV, the mode of the total ion current registration was used for the analysis. Separation was performed on an HP-INNOWAX capillary column (30 m × 250 μm × 0.50 μm). The stationary phase was polyethylene glycol. The mobile phase was helium; gas flow rate was 1 ml/min. Temperature of the evaporator was 250 °C, temperature of the ion source was 230 °C, temperature of the detector was 150 °C, and temperature of the line connecting the chromatograph with the mass spectrometer was 280 °C. Scan range was 41–450 amu. The volume of the injected sample was 1 μl, the flow separation was 5:1. Chromatography was carried out in isothermal mode at 200 °C. To identify the peaks of FA methyl esters, methyl ester standards (Sigma-Aldrich) and mass spectrometry using the NIST 05 mass spectrum library (Ozolina et al., 2017) were used. The content of individual fatty acids was calculated as a percentage of the total amount of fatty acids and divided into groups: saturated (SFA), monounsaturated (MUFA), polyunsaturated (PUFA), saturated iso- and anteiso- methyl branched fatty acids (BFA) (Rodrigues, de Carvalho, 2015).

The significance of differences in biofilm formation and the quantitative content of fatty acids were assessed using the nonparametric Kruskal–Wallis test with Dunnett’s correction (Glantz, 1991). All calculations were performed using the RStudio software.

## Results and discussion

**Fig. 1.**
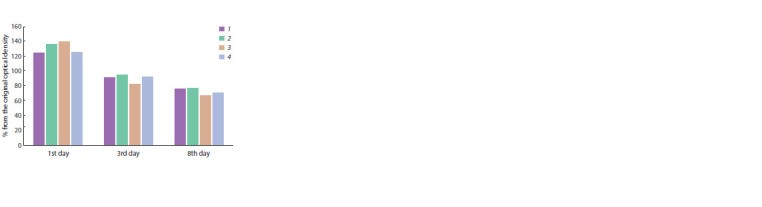
Optical density of R. qingshengii VKM Ac-2784D cell suspension relative to control, %, on the first, third and eighth days of cultivation. Here and in the Fig. 2: 1 – GBSS; 2 – GBSS with 0.03 g/l colchicine; 3 – GBSS with 0.01 g/l colchicine; 4 – GBSS with 0.03 g/l nystatin.

**Fig. 2.**
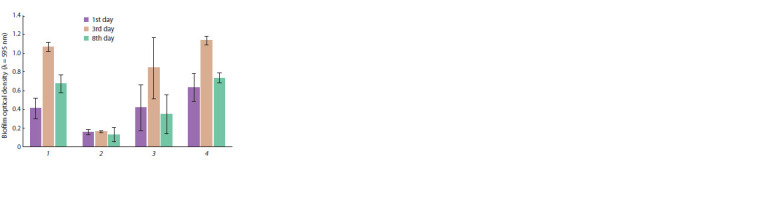
Optical density of biofilm R. qingshengii VKM Ac-2784D on the first, third and eighth days of cultivation.

It was found that the MIC of colchicine for R. qingshengii VKM Ac-2784D is 0.02 g/l. Therefore, in further experiments, the concentrations below and above this value, i. e. 0.01 and 0.03 g/l, respectively, were used. The selected concentrations of colchicine and nystatin did not strongly affect the growth of Rhodococcus (Fig. 1). At the same time, it was established that colchicine at a concentration of 0.03 g/l significantly inhibited the formation of a biofilm at all stages of the experiment, while at a concentration of 0.01 g/l a divergent effect was observed. On the first day, nystatin stimulated the formation of a biofilm, whilst on the third and eighth days of cultivation, its effect was comparable to the control (Fig. 2).

**Fig. 3.**
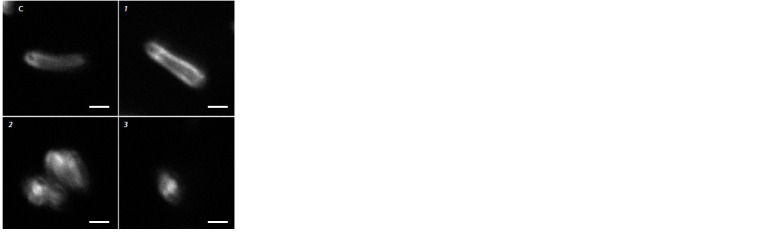
Cell morphology of R. qingshengii VKM Ac-2784D, cultivated for 24 hours in buffered saline with glucose (5 g/L) (C) and after adding 0.03 g/l nystatin (1), 0.01 g/l colchicine (2), 0.03 g/l colchicine (3). Stained with laurdan. Magnification × 600.

The cultivation of R. qingshengii VKM Ac-2784D in the presence of colchicine for a day essentially changed the cell morphology: the cells acquired a more rounded shape (Table 1). Moreover, the intracellular content became heterogeneous (Fig. 3), which is consistent with the results obtained for Bacillus megaterium (Dubey et al., 2011). The shape of Rhodococcus cells under the action of nystatin remained intact.

**Table 1.**
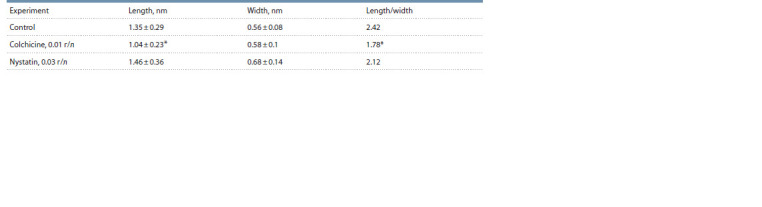
Cell sizes of R. qingshengii VKM Ac-2784D after cultivation for a day under control conditions and in the presence of the studied compounds * p < 0.05.

The changes in cell morphology are usually accompanied by structural and functional rearrangement of their cell membranes (de Carvalho et al., 2014). Specifically, the degree of saturation of FA, their length, as well as the amount of branched fatty acids are altered.

**Fig. 4.**
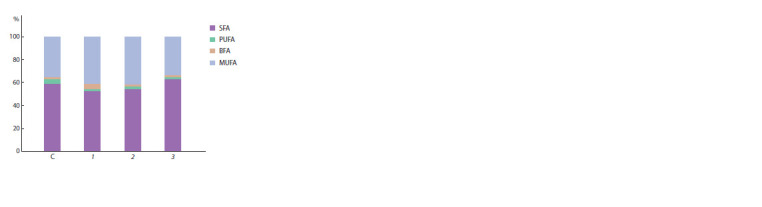
Relative content of the main groups of fatty acids during cultivation for a day in buffered saline with glucose (5 g/l) (C) and with the introduction of 0.03 g/l nystatin (1), 0.01 g/l colchicine (2), 0.03 g/l colchicine (3). SFA – saturated fatty acids; PUFA – polyunsaturated fatty acids; MUFA – monounsaturated fatty acids; BFA –saturated iso- and anteiso-methyl branched fatty acids.

Under the control conditions, the Rhodococcus cell membranes mainly contained palmitic, stearic, and oleic acids (Table 2). The ratio of saturated to monounsaturated fatty acids was 1.64. The content of polyunsaturated and branched acids was small (4.41 and 0.84 %, respectively). Colchicine at a concentration of 0.01 g/l changed the ratio of saturated and monounsaturated FA (1.29) in favor of the latter, and simultaneously reduced the amount of polyunsaturated FA. At the same time, the number of longchain FA C20:1(n-9), C21:0 and C22:0 increased. With 0.03 g/l of colchicine, the relative content of saturated and branched FA increased (Fig. 4), while the ratio of UFA to MUFA reached 1.89. All this indicates the rearrangement of R. qingshengii VKM Ac- 2784D membrane after the introduction of colchicine into the cultivation medium. Interestingly, different concentrations of colchicine had an opposite effect on the composition of cell membrane FA. This is probably due to various degrees of regulatory systems disorder. The addition of nystatin, a compound that fairly increases the membrane fluidity, led to a higher content of unsaturated and branched FA, substantially lower amount of palmitic FA, higher concentration of oleic FA.

**Table 2.**
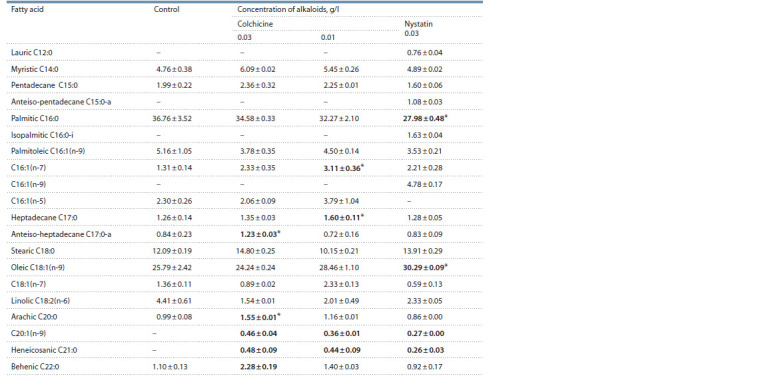
The composition of FA in the membrane cells of R. qingshengii VKM Ac-2784D cultivated for a day in GBSS with colchicine (0.03 and 0.01 g/l) and nystatin (0.03 g/l) * p < 0.05.

The fluidity or microviscosity of membranes is an integral index that depends on lipid saturation and content of sterols or proteins. Therefore, further we focused our efforts on the evaluation of the colchicine and nystatin effect on the orderliness of the lipid phase of R. qingshengii VKM Ac-2784D membrane. For this purpose, the laurdan fluorescence GP index was used, which can vary from –1 to +1. Its negative values correspond to lower microviscosity (higherfluidity) of the cell membrane (Nurminsky et al., 2015) (see Materials and Methods).

**Fig. 5.**
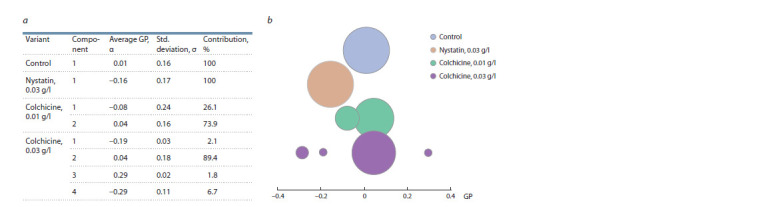
Influence of nystatin and colchicine on the parameters of the components of the distributions of GP values of R. qingshengii VKM Ac-2784D membranes (a) and comparison of the components on the bubble diagram (b); the area of the circle reflects the contribution of each component, n = 10–26.

The fitting of experimental distributions of bacterial membrane GP values permitted to find from one to four components under the action of nystatin and colchicine (Fig. 5). In all variants, the most significant component characterizes the liquid-disordered regions of the membrane (α (average GP values): –0.16–0.04, contribution: 73.9–100 %). The sterol- binding agent nystatin shifted GP towards a decrease in the orderliness of the membranes (α: –0.16, contribution: 100 %), which corresponds to the known mechanism of action of this antibiotic on the membranes of eu- and prokaryotes (Efimova et al., 2014). Colchicine, on the contrary, increased the orderliness of the membranes: a of the most significant components shifted, although slightly, towards positive values compared to the control in both concentrations (α: 0.04, contribution: 73.9–89.4 %), which agrees with the observed increase in the amount of saturated FA. However, this significantly expanded the data scattering, and the number of components reached 2 (in the variant with 0.01 mg/ml) and 4 (in the variant with 0.03 mg/ml). Minor components corresponded to more densely packed regions of the membranes (α: 0.29, contribution: 1.8 %) or, conversely, to less densely packed ones (α: –0.29, contribution: 6.7 %).

## Conclusion

In conclusion, colchicine in the composition of GBSS at concentrations of 0.01 and 0.03 g/l did not significantly affect the survival of R. qingshengii VKM Ac-2784D, but strongly inhibited the formation of a biofilm. Rhodococcus cells cultivated for 24 hours in GBSS with colchicine acquired a rounded shape. With 0.01 g/l of colchicine, the content of C16:1(n-7), C17:0, C20:1(n-9) and C21:0 FA acids increased.

The membrane microviscosity of individual cells is distributed from the lowest to the highest GP values, which indicates a variety of adaptive responses to this alkaloid. At a higher concentration of colchicine (0.03 g/l) in the cell membranes of R. qingshengii VKM Ac-2784D, the content of saturated fatty acids increased, while the amount of branched fatty acids reduced. This enhanced the membrane microviscosity that was confirmed by the values of laurdan fluorescence GP. These data testify to an adaptive rearrangement of the cell membrane under the action of the studied alkaloid, which is consistent with the results obtained by other authors (Wang et al., 2020). This may be a reason of the negative effect of colchicine on the formation of R. qingshengii VKM Ac-2784D biofilms

## Conflict of interest

The authors declare no conflict of interest.
